# Factors impacting the development of empathy in healthcare students towards people with dementia during undergraduate education: a mixed‐methods study

**DOI:** 10.1002/alz70858_102133

**Published:** 2025-12-25

**Authors:** Yvonne Feeney, Stephanie Daley, Nicolas Farina, Molly Hebditch, Sube Banerjee

**Affiliations:** ^1^ Brighton and Sussex Medical School, Brighton, United Kingdom; ^2^ University of Plymouth, Plymouth, United Kingdom; ^3^ University of Nottingham, Nottingham, United Kingdom

## Abstract

To deliver person‐centred care to people with dementia, healthcare professionals need to have the capacity to practice with empathy. Defined as an ability to resonate with the feelings of another and act on that understanding (Slater et al, 2022), higher empathy leads to better patient relationships, reduced burnout, and recognition of personhood (Derkson et al, 2013). As the prevalence of dementia increases globally (ADI, 2023), the healthcare professions must be equipped with the skills to provide empathetic care to people with dementia. However, empathy can decline in healthcare students during their educational years (Hojat et al, 2023). This is a concern as people with dementia frequently report episodes of care that lack empathy. Therefore, an understanding of the factors that impact empathy in students towards people with dementia is needed to improve future educational approaches.

Using a sequential design, a longitudinal mixed‐methods study was completed to understand the factors that impacted empathy towards people with dementia. First, a grounded theory study was completed. Repeated semi‐structured interviews were undertaken at two timepoints with ten nursing, ten physiotherapy, and ten medical students, and data was analysed using constant comparison techniques. Next, a quantitative study, using secondary data analysis was completed on paired survey data that measured student empathy, attitudes, and knowledge of dementia (*n* = 1,611). Regression analysis was used to test the factors that impacted empathy scores over time.

Findings showed that student capacity for empathy towards people with dementia was influenced by the interactions between their moral values and principles, their experiences during clinical exposure, shifting ideals, and the resulting impact on their emotional self‐efficacy. Unique challenges to their empathy included difficulty resonating with the lived experience of dementia, communication barriers, role model influence, emotional distress, and their own levels of confidence. Quantitative findings showed that higher levels of knowledge, comfort, and person‐centredness towards dementia were significant predictors of empathy.

The findings suggest that a multimodal approach to address student, educational, and systemic factors are needed to enhance empathy during the undergraduate years. Improving student confidence and increasing knowledge, comfort, and person‐centredness towards dementia should be prioritised in education to enhance empathy.

